# The Diophantine Equation 8^*x*^ + *p* ^*y*^ = *z* ^2^

**DOI:** 10.1155/2015/306590

**Published:** 2015-01-14

**Authors:** Lan Qi, Xiaoxue Li

**Affiliations:** ^1^College of Mathematics and Statistics, Yulin University, Yulin, Shaanxi 719000, China; ^2^School of Mathematics, Northwest University, Xi'an, Shaanxi 710127, China

## Abstract

Let *p* be a fixed odd prime. Using certain results of exponential Diophantine equations, we prove that (i) if *p* ≡ ±3(mod  8), then the equation 8^*x*^ + *p* ^*y*^ = *z* ^2^ has no positive integer solutions (*x*, *y*, *z*); (ii) if *p* ≡ 7(mod  8), then the equation has only the solutions (*p*, *x*, *y*, *z*) = (2^*q*^ − 1, (1/3)(*q* + 2), 2, 2^*q*^ + 1), where *q* is an odd prime with *q* ≡ 1(mod  3); (iii) if *p* ≡ 1(mod  8) and *p* ≠ 17, then the equation has at most two positive integer solutions (*x*, *y*, *z*).

## 1. Introduction

Let *Z*, *N* be the sets of all integers and positive integers, respectively. Let *p* be a fixed odd prime. Recently, the solutions (*x*, *y*, *z*) of the equation

(1) $$\begin{array}{c} 8^{x}+p^{y}=z^{2},\;x,y,z\in N \end{array}$$

were determined in the following cases:

1. (Sroysang [1]) if *p* = 19, then (1) has no solutions;
2. (Sroysang [2]) if *p* = 13, then (1) has no solutions;
3. (Rabago [3]) if *p* = 17, then (1) has only the solutions (*x*, *y*, *z*) = (1,1, 5), (2,1, 9), and (3,1, 23).

In this paper, using certain results of exponential Diophantine equations, we prove a general result as follows.

**Theorem 1 .**

If *p* ≡ ±3(mod⁡  8), then (1) has no solutions (*x*, *y*, *z*). If *p* ≡ 7(mod⁡  8), then (1) has only the solutions

(2) $$\begin{array}{c} \left(p,x,y,z\right)=\left(2^{q}-1,\frac{q+2}{3},2,2^{q}+1\right), \end{array}$$

where *q* is an odd prime with *q* ≡ 1(mod⁡  3).

If *p* ≡ 1(mod⁡  8) and *p* ≠ 17, then (1) has at most two solutions (*x*, *y*, *z*).

Obviously, the above theorem contains the results of [1, 2]. Finally, we propose the following conjecture.

**Conjecture 2 .**

If *p* ≠ 17, then (1) has at most one solution (*x*, *y*, *z*).

## 2. Preliminaries

**Lemma 3 .**

If 2^*n*^ − 1 is a prime, where *n* is a positive integer, then *n* must be a prime.

**Proof**

See Theorem 1.10.1 of [4].

**Lemma 4 .**

If *p* is an odd prime with *p* ≡ 1(mod⁡  4), then the equation

(3) $$\begin{array}{c} u^{2}-pv^{2}=-1,\;u,v\in N \end{array}$$

has solutions (*u*, *v*).

**Proof**

See Section 8.1 of [5].

**Lemma 5 .**

The equation

(4) X2−2m=Yn, X,Y,m,n∈N,  gcd⁡X,Y=1,  Y>1,                m>1, n>2

has only the solution (*X*, *Y*, *m*, *n*) = (71,17,7, 3).

**Proof**

See Theorem 8.4 of [6].

**Lemma 6 .**

Let *D* be a fixed odd positive integer. If the equation

(5) $$\begin{array}{c} u^{2}-Dv^{2}=-1,\;u,v\in N \end{array}$$

has solutions (*u*, *v*), then the equation

(6) $$\begin{array}{c} X^{2}-D=2^{n},\;X,n\in N,\;\;n>2 \end{array}$$

has at most two solutions (*X*, *n*), except the following cases:

- *D* = 2^2*r*^ − 3 · 2^*r*+1^ + 1, (*X*, *n*) = (2^*r*^ − 3,3), (2^*r*^ − 1, *r* + 2), (2^*r*^ + 1, *r* + 3), and (3 · 2^*r*^ − 1,2*r* + 3), where *r* is a positive integer with *r* ≥ 3;
- *D* = ((1/3)(2^2*r*+1^ − 17))^2^ − 32, (*X*, *n*) = ((1/3)(2^2*r*+1^ − 17), 5), (1/3)(2^2*r*+1^ + 1,2*r* + 3), and ((1/3)(17 · 2^2*r*+1^ − 1), 4*r* + 7), where *r* is a positive integer with *r* ≥ 3;
- *D* = 2^2*r*_1_^ + 2^2*r*_2_^ − 2^*r*_1_+*r*_2_+1^ − 2^*r*_1_+1^ − 2^*r*_2_+1^ + 1, (*X*, *n*) = (2^*r*_2_^ − 2^*r*_1_^ − 1, *r*_1_ + 2), (2^*r*_2_^ − 2^*r*_1_^ + 1, *r*_2_ + 2), and (2^*r*_2_^ + 2^*r*_1_^ − 1, *r*_1_ + *r*_2_ + 2), where *r*_1_, *r*_2_ are positive integers with *r*_2_ > *r*_1_ + 1 > 2.

**Proof**

See [7].

**Lemma 7 .**

If *D* is an odd prime and *D* belongs to the exceptional case (i) of Lemma 6, then *D* = 17.

**Proof**

We now assume that *D* is an odd prime with *D* = 2^2*r*^ − 3 · 2^*r*+1^ + 1. Then we have

(7) $$\begin{array}{c} {\left(2^{r}-1\right)}^{2}-2^{r+2}=D, \end{array}$$

(8) $$\begin{array}{c} {\left(2^{r}+1\right)}^{2}-2^{r+3}=D. \end{array}$$

If 2∣*r*, since *r* ≥ 3, then *r* ≥ 4, and by (7), we have

(9) $$\begin{array}{c} \left(2^{r}-1\right)+2^{r/2+1}=D,\;\;\left(2^{r}-1\right)-2^{r/2+1}=1. \end{array}$$

But, by the second equality of (9), we get 1 ≡ (2^*r*^ − 1) − 2^*r*/2+1^ ≡ −1(mod⁡  8), a contradiction.

If 2∤*r*, then from (8) we get

(10) $$\begin{array}{c} \left(2^{r}+1\right)+2^{(r+3)/2}=D,\;\;\left(2^{r}+1\right)-2^{(r+3)/2}=1. \end{array}$$

Further, by the second equality of (10), we have 2^*r*^ = 2^(*r*+3)/2^, *r* = 3, and *D* = 17. Thus, the lemma is proved.

**Lemma 8 .**

If *D* is an odd prime and *D* belongs to the exceptional case (iii) of Lemma 6, then *D* = 17.

**Proof**

Using the same method as in the proof of Lemma 7, we can obtain this lemma without any difficulty.

**Lemma 9 .**

If *D* belongs to the exceptional case (ii), then (6) has at most one solution (*X*, *n*) with 3∣*n*.

**Proof**

Notice that, for any positive integer *r*, there exists at most one number of 5, 2*r* + 3, and 4*r* + 7 which is a multiple of 3. Thus, by Lemma 6, the lemma is proved.

**Lemma 10 .**

The equation

(11) $$\begin{array}{c} X^{m}-Y^{n}=1,\;X,Y,m,n\in N,\;\;\min\left\{X,Y,m,n\right\}>1 \end{array}$$

has only the solution (*X*, *Y*, *m*, *n*) = (3,2, 2,3).

**Proof**

See [8].

## 3. Proof of Theorem

We now assume that (*x*, *y*, *z*) is a solution of (1). Then we have gcd(2*p*, *z*) = 1.

If 2∣*y*, since gcd(*z* + *p* ^*y*/2^, *z* − *p* ^*y*/2^) = 2, then from (1) we get

(12) $$\begin{array}{c} z+p^{y/2}=2^{3x-1},\;\;z-p^{y/2}=2, \end{array}$$

where we obtain

(13) $$\begin{array}{c} z=2^{3x-2}+1, \end{array}$$

(14) $$\begin{array}{c} p^{y/2}=2^{3x-2}-1. \end{array}$$

Since *p* > 1, applying Lemma 10 to (14), we get

(15) $$\begin{array}{c} y=2,\;\;p=2^{3x-2}-1. \end{array}$$

Further, by Lemma 3, we see from the second equality of (15) that

(16) $$\begin{array}{c} p=2^{q}-1,\;\;q=3x-2 \end{array}$$

is an odd prime with *q* ≡ 1(mod⁡3).

Therefore, by (13), (15), and (16), we obtain the solutions given in (2).

Obviously, if *p* satisfies (2), then *p* ≡ 7(mod⁡  8). Otherwise, since 2∤*y*, we see from (1) that *p* ≡ *p* ^*y*^ ≡ *z* ^2^ − 8^*x*^ ≡ 1(mod⁡  8). It implies that if *p* ≡ ±3(mod⁡  8), then (1) has no solutions (*x*, *y*, *z*). If *p* ≡ 7(mod⁡  8), then (1) has only the solutions (2).

Here and below, we consider the remaining cases that *p* ≡ 1(mod⁡  8). By the above analysis, we have 2∤*y*. If *y* > 1, then *y* ≥ 3 and (4) has the solution (*X*, *Y*, *m*, *n*) = (*z*, *p*, 3*x*, *y*) with 3∣*m*. But, by Lemma 5, it is impossible. Therefore, we have

(17) $$\begin{array}{c} y=1. \end{array}$$

Substituting (17) into (1), the equation

(18) $$\begin{array}{c} X^{2}-p=2^{n},\;X,n\in N,\;\;n>2 \end{array}$$

has the solution (*X*, *n*) = (*z*, 3*x*) with 3∣*n*. Since *p* ≡ 1(mod⁡  8), by Lemma 4, (3) has solutions (*u*, *v*). Therefore, by Lemmas 6–9, (1) has at most two solutions (*x*, *y*, *z*). Thus, the theorem is proved.
